# High-Switching-Ratio Photodetectors Based on Perovskite CH_3_NH_3_PbI_3_ Nanowires

**DOI:** 10.3390/nano8050318

**Published:** 2018-05-10

**Authors:** Xin Zhang, Caichi Liu, Gang Ren, Shiyun Li, Chenghao Bi, Qiuyan Hao, Hui Liu

**Affiliations:** 1 School of Materials Science and Engineering, Hebei University of Technology, Tianjin 300132, China; 18202669220@163.com (X.Z.); ccliu@hebut.edu.cn (C.L.); 18222902706@163.com (G.R.); 13682087532@163.com (S.L.); chenghao_bi@163.com (C.B.); 2 School of Xingtai Polytechnic College, Xingtai 054035, China

**Keywords:** perovskite, CH_3_NH_3_PbI_3_ nanowires, spincoating, photodetectors

## Abstract

Hybrid organic-inorganic perovskite materials have attracted extensive attention due to their impressive performance in photovoltaic devices. One-dimensional perovskite CH_3_NH_3_PbI_3_ nanomaterials, possessing unique structural features such as large surface-to-volume ratio, anisotropic geometry and quantum confinement, may have excellent optoelectronic properties, which could be utilized to fabricate high-performance photodetectors. However, in comparison to CH_3_NH_3_PbI_3_ thin films, reports on the fabrication of CH_3_NH_3_PbI_3_ nanowires for optoelectrical application are rather limited. Herein, a two-step spin-coating process has been utilized to fabricate pure-phase and single-crystalline CH_3_NH_3_PbI_3_ nanowires on a substrate without mesoporous TiO_2_ or Al_2_O_3_. The size and density of CH_3_NH_3_PbI_3_ nanowires can be easily controlled by changing the PbI_2_ precursor concentration. The as-prepared CH_3_NH_3_PbI_3_ nanowires are utilized to fabricate photodetectors, which exhibit a fairly high switching ratio of ~600, a responsivity of 55 mA/W, and a normalized detectivity of 0.5 × 10^11^ jones under 532 nm light illumination (40 mW/cm^2^) at a very low bias voltage of 0.1 V. The as-prepared perovskite CH_3_NH_3_PbI_3_ nanowires with excellent optoelectronic properties are regarded to be a potential candidate for high-performance photodetector application.

## 1. Introduction

Photodetectors, which convert incident light signals into electronic signals, are important devices for application in a wide range of civilian and military fields, including optical communications, environmental sensors, medical analysis, missile launch detection, and so forth [1,2,3]. The crucial characteristics of high-performance photodetectors for practical application include wide spectral response, sensitivity, high switching ratio, fast response, large detectivity and easy fabrication. Many semiconductor nanomaterials, such as ZnO, Si, CdS, PbS, CdHgTe, have been applied in photodetectors that can detect the light ranged from UV to infrared region [4,5,6]. Recently, hybrid organic-inorganic perovskite materials, such as CH_3_NH_3_PbI_3_ (MAPbI_3_), have been extensively studied and applied in solar cells [7,8,9], LEDs [10,11] and laser devices [12] due to their distinct photoelectric properties, which include high absorption coefficient, direct and tunable bandgap, weak exciton binding energy, high carrier mobility and long carrier-diffusion lengths.

In the past few decades, one-dimensional (1D) semiconductor nanomaterials have been considered the most promising candidates for achieving high-performance photodetectors with high switching ratio (*SR*), large responsivity (*R*_λ_), fast response speed and excellent stability, which can be attributed to their large surface-to-volume ratio, anisotropic geometry and quantum confinement in two dimensions [13]. Therefore, the fabrication of 1D perovskite MAPbI_3_ nanomaterials has attracted significant interest from researchers. Several methods have been successfully utilized to prepare MAPbI_3_ nanowires or microwires, including slip-coating method [14], dissolution-recrystallization process [15], template guide growth technology [16,17], inkjet printing method [18], and so on. However, in comparison to MAPbI_3_ thin films [19,20,21], the reports about the fabrication of MAPbI_3_ nanowires for application in optoelectrical application [22,23] are rather limited. In addition, some reports also indicate that single-crystalline perovskite nanowires have very low defect levels and impressive optoelectrical properties [24,25,26], which are comparable or even better than their large single-crystal counterpart. Therefore, developing a high-efficiency photodetector based on single-crystalline perovskite nanowires is of great significance.

Recently, a two-step spin-coating process, which was firstly reported by Park’s group, was utilized to synthesize CH_3_NH_3_PbI_3_ nanowires on a mesoporous TiO_2_ or Al_2_O_3_ substrate for application in perovskite solar cell, with a power conversion efficiency (PCE) of 14.71% at standard AM (Path-length through the atmosphere relative to vertical thickness of the atmosphere) 1.5 G solar illumination [27]. However, as far as we know, scarcely any works have been reported that use the two-step spin-coating process to prepare single MAPbI_3_ nanowires on a substrate without mesoporous TiO_2_ or Al_2_O_3_ and apply them in photodetectors. In this work, we improved the two-step spin-coating process to fabricate pure-phase and single-crystalline MAPbI_3_ nanowires with various densities and sizes on a SiO_2_/Si substrate just by changing the PbI_2_ precursor concentration. The as-prepared MAPbI_3_ nanowires were used to fabricate photodetectors that exhibited a fairly high switching ratio of ~600, responsivity of 55 mA/W and normalized detectivity of 0.5 × 10^11^ jones under 532 nm light illumination (40 mW/cm^2^) at a very low bias voltage of 0.1 V. To the best of our knowledge, the high switching ratio is one of the best results among previously reported perovskite-based photodetectors [15,19,20,21,22,23,28]. The MAPbI_3_ nanowires with excellent optoelectronic properties may be an ideal choice for high-performance photodetectors.

## 2. Materials and Methods 

### 2.1. Materials and Chemicals

Lead iodide (PbI_2_, 99.9%, Aladdin, Shanghai, China), *N*,*N*-dimethylformamide (DMF, 99%, Aladdin), methylamine (CH_3_NH_2_, 33% in absolute methanol, Aladdin), hydroiodic acid (HI, 58 wt % in water, Aladdin), Lead iodide (C_2_H_5_OH, 99.9%, Aladdin), Isopropanol (C_3_H_8_O, 99.9%, Aladdin), Ethyl ether (C_4_H_10_O, 99.9%, Aladdin). All chemicals were used as received.

### 2.2. Preparation of CH_3_NH_3_I (MAI)

CH_3_NH_3_I was prepared according to the reported process with some modifications [17]. Typically, 24 mL methylamine (CH_3_NH_2_) (33 wt % in absolute methanol, Aladdin, China) and 10 mL of hydroiodic acid (HI) (58 wt % in water, Aladdin, Shanghai, China) were in a 250 mL round-bottom flask at 0 °C for 2 h with stirring to synthesize. The precipitate was collected by evaporating the solvents on a rotary evaporator at 50 °C. The MAI product was washed and precipitated with the addition of the absolute ethanol and diethyl ether for three times, respectively. The solid was collected and dried at 60 °C in a vacuum oven for 24 h.

### 2.3. Preparation of CH_3_NH_3_PbI_3_ (MAPbI_3_) Nanowires

The SiO_2_/Si substrates were cleaned in an ultrasonic bath with acetone, isopropyl and ethanol for 5 min, respectively, and dried with clean N_2_ for further use. MAPbI_3_ nanowires were formed using the two-step spin-coating process. Firstly, to deposit PbI_2_ precursor layer, PbI_2_ precursor solution with different concentrations of 0.5 M, 0.4 M, 0.3 M and 0.2 M, respectively, was prepared by dissolving a certain amount of PbI_2_ in 2 mL of DMF and stirring in room temperature. Then, 20 μL of PbI_2_ precursor solution was loaded on the substrate for 10 s, followed by spinning at 2000 rpm for 5 s and 6000 rpm for 5 s. Secondly, 17.5 mg MAI powders were poured in 5 mL isopropanol (IPA), including 5 μL of DMF, and then stirred at room temperature until dissolved. 200 μL of the MAI-IPA solution was loaded on the PbI_2_-coated substrate for 40 s, followed by spinning at 4000 rpm for 20 s and drying at 100 °C in an oven for 5 min. Finally, the final MAPbI_3_ nanowires with different size and density can be obtained. All of the process were in air.

### 2.4. Device Fabrication

The interdigital Au electrodes with interfinger distance of 4 μm and length of 1000 μm were fabricated on SiO_2_/Si substrates using the conventional lithography technique. The abovementioned MAPbI_3_ nanowires synthesized with different concentrations of PbI_2_ precursor solution were spin-coated on the interdigitated Au electrodes for further photoelectric characterization.

### 2.5. Structural Characterization

X-ray diffraction (XRD) was detected by Rigaku D/Max 2500 V/PC X-ray powder diffractometer (Hitachi, Tokyo, Japan) with CuKa radiation. FESEM (Rigaku, Tokyo, Japan) morphology and Energy Dispersive X-ray Fluorescence (EDX) analyses were performed using a Hatchi s-4800 field emission scanning electron microscope (Hitachi, Tokyo, Japan). Transmission electron microscopy (TEM) and high-resolution transmission electron microscopy (HRTEM) were performed using a Tecnai G2 F20 field emission transmission electron microscope (FETEM) (Philippe, Amsterdam, The Netherlands). Absorption spectra were recorded by a U-3900 H Spectrophotometer with optics integrating sphere (Hitachi, Tokyo, Japan). Fluorescence spectra were recorded with an F-7000 FL spectrofluorometer (Japan High-tech Corporation, Tokyo, Japan). Photoresponse characterization were done using a digital sourcemeter (keithley 2400) and a monochromatic light source (Bo Feilai Technology Co., Ltd., Beijing, China).

## 3. Results

**Figure 1:**
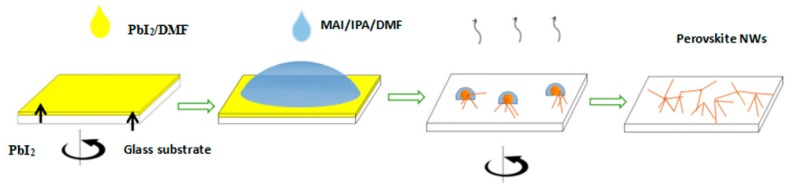
The schematic illustration of the two-step spin-coating process. Firstly, PbI_2_-DMF precursor solution was spin-coated onto a SiO_2_/Si substrate to form PbI_2_ thin films, followed by dripping the MAI-IPA solution including DMF solution on the films. Secondly, the substrate was spun again to evaporate the solvent. Finally, the obtained films were annealed in an oven to form MAPbI_3_ nanowires.

Figure 1 presents the two-step spin-coating process scheme for synthesizing perovskite nanowires. Firstly, PbI_2_-DMF precursor solution with different concentrations was spin coated onto a SiO_2_/Si substrate to form PbI_2_ thin films, which was called the first spin-coating stage. Secondly, 200 μL MAI-IPA solution (17.5 mg MAI/5 mL IPA), including 5 μL DMF solution, was loaded on the PbI_2_ thin films for 10 s followed by spin coating, which was called the second spin-coating stage. Then, the obtained films were annealed in an oven. Finally, the perovskite MAPbI_3_ nanowires were obtained.

**Figure 2:**
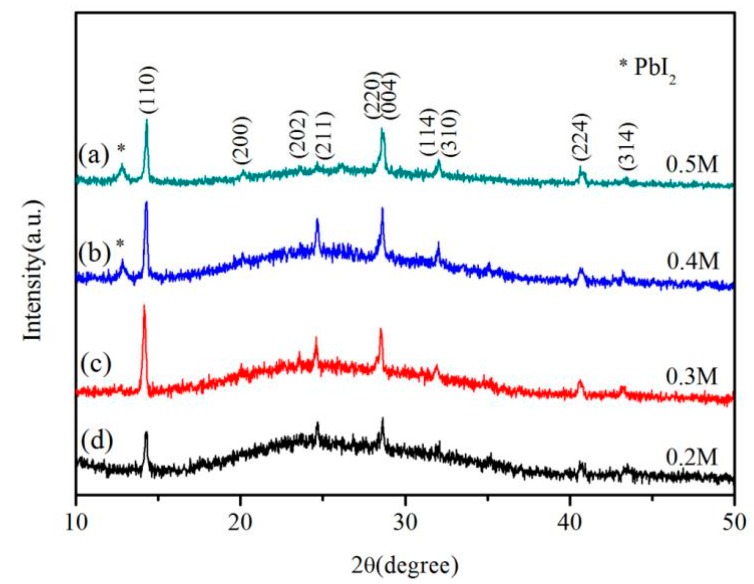
XRD patterns of perovskite nanowires synthesized with different PbI_2_ precursor concentration of (**a**) 0.5 ML; (**b**) 0.4 M; (**c**) 0.3 M and (**d**) 0.2 M.

**Figure 3:**
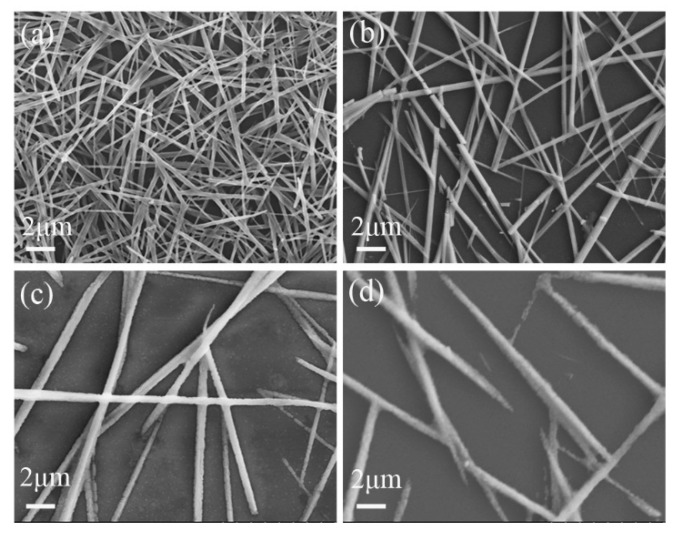
SEM (Transmission electron microscopy images) of perovskite nanowires synthesized with different PbI_2_ precursor concentration of (**a**) 0.5 M; (**b**) 0.4 M; (**c**) 0.3 M and (**d**) 0.2 M.

Figure 2 shows the XRD patterns of the perovskite nanowires synthesized with different PbI_2_ concentration of 0.5 M, 0.4 M, 0.3 M and 0.2 M. The main diffraction peaks at 2θ = 14.20°, 24.49°, 28.32°, 28.49°, 31.82° and 40.79° (Figure 2a,b) correspond to (110), (211), (004), (220), (310) and (224) planes of the tetragonal perovskite MAPbI_3_, which are in agreement with the references [15,27,29,30]. In addition, the weak diffraction peak at 2θ = 12.7° shown in Figure 2a,b can be indexed to the (001) plane of hexagonal PbI_2_ (JCPDS. No. 07-0235), indicating that a small amount of PbI_2_ was present in the products. Further decreasing the PbI_2_ concentration to 0.3 M and 0.2 M (Figure 2c,d), the products are composed of pure-phase tetragonal perovskite MAPbI_3_. Furthermore, the PbI_2_ concentration will also affect the size and density of the synthesized MAPbI_3_ nanowires, which is shown in Figure 3. It can be clearly seen that, with the decrease of PbI_2_ concentration from 0.5 M to 0.2 M, the size of MAPbI_3_ nanowires will increase, with the average diameter increasing from 180 nm to 850 nm and the average length increasing from several microns to dozens of microns, while the density of MAPbI_3_ nanowires decreases. In addition, the size distribution of the MAPbI_3_ nanowires become more broaden with the decrease of PbI_2_ concentration in the first spin-coating step.

**Figure 4:**
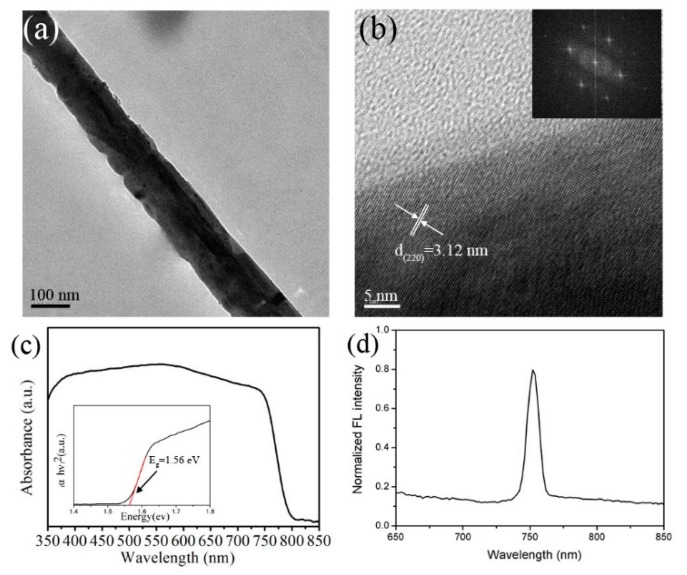
The morphology and optical properties characterization of the synthesized perovskite MAPbI_3_ nanowires. (**a**) TEM image; (**b**) HRTEM image; (**c**) UV-vis absorbance spectra and (**d**) fluorescence spectra.

As is well known, DMF is a benign solvent, while IPA is a poor solvent for PbI_2_. A small amount of DMF in IPA solution will dissolve PbI_2_ after dropping it on PbI_2_ precursor films, which will form a liquid cluster containing dissolved PbI_2_ and MAI molecules [27]. During the secondary spin-coating stage, the rapid evaporation of solvent will lead to sudden supersaturation and form quick nucleation of perovskite MAPbI_3_. Furthermore, the tetragonal perovskite MAPbI_3_ has a tendency to form nanowires by self-assembly of particles [15], which is also confirmed in Figure S1. Along with decreasing the PbI_2_ concentration during the first spin-coating process, the supersaturation level of perovskite MAPbI_3_ in solution will also decrease, leading to fewer nucleation centers and lower density of nanowires. Due to the smaller number of nuclei, fewer monomers are exhausted at the stage of nucleation, which can be utilized to encourage each nucleus to increase in size at the stage of crystal growth, leading to a larger size of MAPbI_3_ nanowires. This is consistent with the SEM (Transmission electron microscopy images) images in Figure 3. Therefore, the supersaturation level of perovskite MAPbI_3_ in solution is a crucial factor in influencing the final morphologies of perovskite nanowires. In addition to PbI_2_ concentration in the first spin-coating stage, the DMF concentration and MAI concentration in IPA in the secondary spin-coating stage can also effectively influence the kinetics of nanowire growth. It’s easy to conclude that, with the decrease of DMF concentration and the increase of MAI concentration in IPA, the supersaturation level of monomer solution will increase and generate more nucleation centers, leading to higher density of perovskite MAPbI_3_ nanowires with smaller size, which is consistent with the SEM results in Figures S2 and S3. TEM and HRTEM images of one typical perovskite MAPbI_3_ nanowire are presented in Figure 4a,b. It can be seen that the perovskite nanowire has a uniform diameter, which is shown in Figure 4a. The clear crystalline lattice and identical orientation to the typical nanowire indicate that it’s a single crystal with an interplanar distance of 0.312 nm, which corresponds to the (220) plane of tetragonal perovskite MAPbI_3_ (Figure 4b). The FFT image inserted in Figure 4b also demonstrates that the perovskite nanowire is a single crystal. Figure 4c shows the UV-Vis absorption spectra of the perovskite MAPbI_3_ nanowires. This result indicates that the MAPbI_3_ nanowires exhibit a strong and broad range of light absorption from 350 to 800 nm, which absolutely covers the entire visible light spectrum. The band gap calculated by Tauc’s formula shown in the insert of Figure 4c is about 1.56 eV, which agrees well with the reported perovskite MAPbI_3_ nanowires [27]. In addition, the MAPbI_3_ nanowires display a strong and sharp photoluminescence peak situated at 755 nm, which is almost consistent with the reported literature [27].

**Figure 5:**
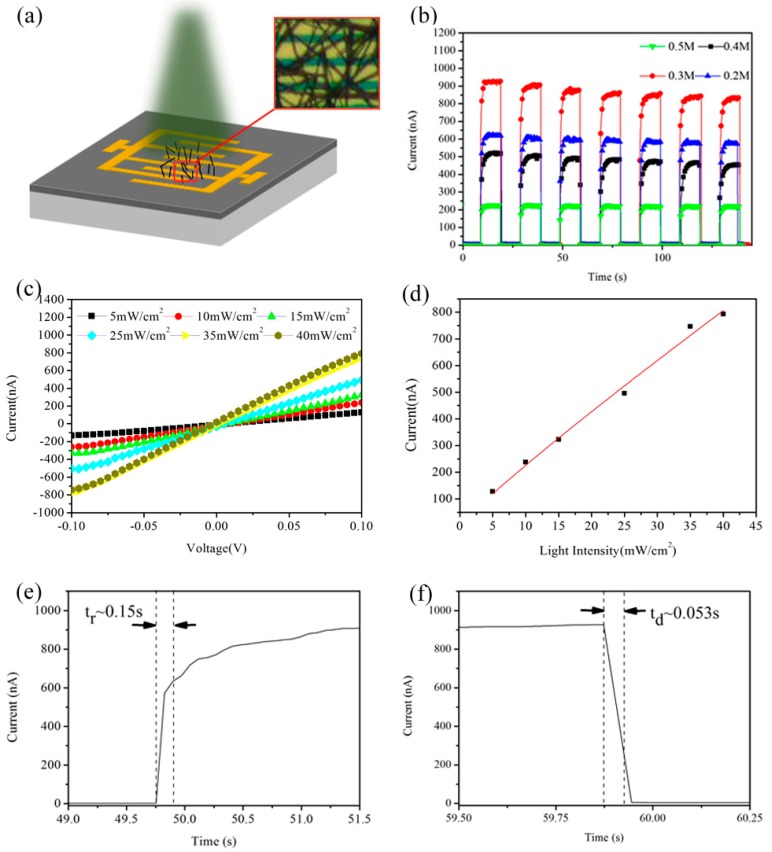
The photoresponsive properties of the photodetector based on MAPbI_3_ nanowires. (**a**) The schematic illustration of a photodetector; (**b**) the I-t curves of the perovskite nanowire photodetector measured under 532 nm light illumination (40 mW/cm^2^) at a low bias voltage of 0.1 V; (**c**) the I-V(Current curve with voltage transformation) curves measured under 532 nm light illumination with different light intensity of 5 mW/cm^2^, 10 mW/cm^2^, 15 mW/cm^2^, 25 mW/cm^2^, 35 mW/cm^2^, 40 mW/cm^2^ at a low bias voltage of 0.1 V; (**d**) the photocurrent measured as a function of incident light intensity at a bias voltage of 0.1 V; and (**e**,**f**) the rise and decay times, respectively, for one period of I-V curves displayed in (**b**).

In order to further study the photoresponse properties of the MAPbI_3_ nanowires, a photodetector based on MAPbI_3_ nanowires was fabricated, with a schematic illustration shown in Figure 5a. The interdigital Au electrodes with an interfinger distance of 4 μm and a length of 1000 μm were prepared on SiO_2_/Si substrates using the conventional lithography technique. The pure-phase MAPbI_3_ nanowires synthesized with 0.3 M PbI_2_ precursor solution were spin-coated on the interdigitated electrodes. The key parameters of photodetectors are the switching ratio (*SR*), responsivity (*R*), detectivity (D*) and response speed [31]. The switching ratio is defined as *SR* = ((*I*_p_ − *I*_d_)/*I*_d_), where *I*_p_ is photocurrent, *I*_d_ is dark current. Responsivity can be calculated by *R* = ((*I*_p_ − *I*_d_)/(*P*·*S*). *P* is the light power intensity and *S* is the effective sensitive areas, which are defined as the coverage areas of the interdigital Au electrodes by the MAPbI_3_ nanowires. Considering that the shot noise dominates the total noise in photoconductive photodetectors, normalized detectivity can be given by D* = ((*I*_p_ − *I*_d_)/(*P*(2 e·*I*_d_·*S*)^1/2^)), where D* represents elementary charge. Figure 5b presents the current-time (I-t) curves of the MAPbI_3_-based photodetectors synthesized with different PbI_2_ concentrations of 0.5 M, 0.4 M, 0.3 M and 0.2 M, respectively. Several cycles of “on” (under illumination) and “off” (under dark) states indicate that the four devices have a certain degree of reversibility and stability. The MAPbI_3_ nanowires synthesized with 0.3 M PbI_2_ concentration have the biggest photocurrent among the four nanowires with different size and density, together with a high switching ratio (“on”/“off” current) of ~600 with the dark current of 1.55 nA and the photocurrent of 920 nA under 532 nm illumination with a light intensity of 40 mW/cm^2^ at a very low bias voltage of 0.1 V. The superior photoresponse performance of the MAPbI_3_ nanowires synthesized with 0.3 M concentration may be attributed to the pure phase, high crystalline degree and large length-to-diameter ratio, according to the XRD and SEM results. To the best of our knowledge, the switching ratio of the MAPbI_3_ nanowire photodetectors in this work is one of the best results among previously reported perovskite-based photodetectors including MAPbI_3_ thin films and nanowires [19,20,21,22,23,28], as shown in Table 1.

In addition, a responsivity of 55 mA/w and normalized detectivity of 0.5 × 10^11^ jones are obtained. The excellent photoresponse property may be accounted for by the carrier-trapping mechanism that dominates photoconduction in the one-dimensional nanomaterials [32,33]. Since both the preparation process and the photoelectric characterization of the synthesized perovskite nanowires are exposed in air, amounts of oxygen molecules will chemisorb onto surface dangling bonds and capture the free electron in MAPbI_3_ nanowires under dark conditions, which leads to a low-conductivity depletion layer near the surfaces. On the other hand, under illumination, the photogenerated holes will migrate to the surface and be recombined by the negatively charged oxygen molecules, leaving the unpaired electrons which will increase the conductivity of the MAPbI_3_ nanowires. In order to confirm our assumption, more MAPbI_3_ nanowires were fabricated under Ar with the same procedure and tested in a glove box that was full of Ar. Under the same test conditions, except for the inner atmosphere, the switching ratio of the MAPbI_3_-based photodetector decreased to 35 with a dark current of 22 nA and a photocurrent of 779 nA, as shown in Figure S4. It can be seen that the photocurrent of the device under Ar was almost unchanged, while the dark current increased by more than one order of magnitude, which indicates that the carrier-trapping mechanism is accountable for the improvement of the one-dimensional MAPbI_3_-based photodetector in air.

Figure 5c indicates that the photocurrent obviously increases with the increase in the intensity of incident light, which is attributed to the change in photo-generated carrier concentrations at different incident light densities. The MAPbI_3_-based photodetector exhibits a linear response with the light intensity ranging from 5 mW/cm^2^ to 40 mW/cm^2^ (Figure 5d), indicating that the synthesized MAPbI_3_ nanowire photodetector has a desirable characteristic in terms of its identical responsivity over a wide range of light intensity [34]. The response speed, which includes rise time and decay time, is a critical parameter for evaluating the performance of a photodetector, is defined as the time of starting from turning on the light to reaching 70% of the peak value of photocurrent, or vice versa [18]. From one period of “on/off” states under 538 nm light illumination (40 mW/cm^2^) at 0.1 V, as shown in Figure 5e,f, the rise time and decay time are 0.15 s and 0.053 s, respectively. within comparison to the reported perovskite-based photodetector [15], the rise time is similar, but the decay time is almost 4 times faster, indicating that the perovskite MAPbI_3_ nanowire photodetector has a fast photoresponse speed.

## 4. Conclusions

In summary, an improved two-step spin-coating process was successfully used to fabricate pure-phase and single-crystalline perovskite MAPbI_3_ nanowires. By changing the PbI_2_ precursor concentration, the size and density of MAPbI_3_ nanowires can be easily controlled, whereby the diameter can range from180 nm to 850 nm, and the length can range from several microns to dozens of microns. The as-prepared MAPbI_3_ nanowires were used to fabricate photodetectors, which exhibited a fairly high switching ratio of ~600 under 532 nm light illumination (40 mW/cm^2^) at a very low bias voltage of 0.1 V. This work may provide an effective route for fabricating various kinds of hybrid organic-inorganic perovskite nanowires and the realization of low-cost, solution-processed and high-performance hybrid organic-inorganic perovskite photodetectors.

## Figures and Tables

**Table 1:** Device performance comparison between this work and other MAPbI3-based photodetectors.

Materials	Photocurrent (nA)	Dark Current (nA)	On/Off Ratio	Bias Voltage(V)	**Ref.**
CH_3_NH_3_PbI_3_ single NWs	115	5	23	2	[22]
CH_3_NH_3_PbI_3_ single NWs	0.25	10^−3^	250	1	[23]
CH_3_NH_3_PbI_3_ single NWs	Not Given	Not Given	13	3	[15]
CH_3_NH_3_PbI_3_ thin film	185	5	37	5	[19]
CH_3_NH_3_PbI_3_ thin film	1.75*10^3^	54	324	8	[20]
CH_3_NH_3_PbI_3_ thin film	Not Given	Not Given	23.5	5	[21]
CH_3_NH_3_PbI_3_ single NWs	920	1.55	600	0.1	This work

## Supplementary Materials

The following are available online at http://www.mdpi.com/2079-4991/8/5/318/s1, Figure S1: SEM images of perovskite MAPbI3 nanowires formed by self-assembly particles, Figure S2: SEM images of perovskite MAPbI3 nanowires with DMF volume of (a) 15 μL, (b) 10 μL, (c) 5 μL, Figure S3: SEM images of perovskite MAPbI3 nanowires with MAI concentration in IPA (a) 12.5 mg/5 mL, (b) 22.5 mg/5 mL, (c) 27.5 mg/5 mL, (d) 37.5 mg/5 mg, Figure S4: The I-t curves of devices fabricated under Ar and tested in inner atmosphere.
