# Supplementary material for: High-Switching-Ratio Photodetectors Based on Perovskite CH3NH3PbI3 Nanowires
Source: Nanomaterials (Basel). 2018 May 10;8(5):318. doi: 10.3390/nano8050318 (PMC5977332; doi:10.3390/nano8050318)
Supplement: Supplementary file 1 [file nanomaterials-08-00318-s001.pdf]

nanomaterials

High-Switching-Ratio Photodetectors Based on Perovskite $CH_3NH_3PbI_3$ Nanowires

Xin Zhang [1,2], Caichi Liu [1], Gang Ren [1], Shiyun Li [1], Chenghao Bi [1], Qiuyan Hao [1,*] and Hui Liu [1,*]

[1] School of Materials Science and Engineering, Hebei University of Technology, Tianjin 300132, China; 18202669220@163.com (X.Z.); ccliu@hebut.edu.cn (C.L.); 18222902706@163.com (G.R.); 13682087532@163.com (S.L.); chenghao_bi@163.com (C.B.)
[2] School of Xingtai Polytechnic College, Xingtai 054035, China
* Correspondence: haoqiuyan@hebut.edu.cn (Q.H.); liuhuihebut@163.com (H.L.); Tel.: +86-22-265-8275 (Q.H. & H.L.)

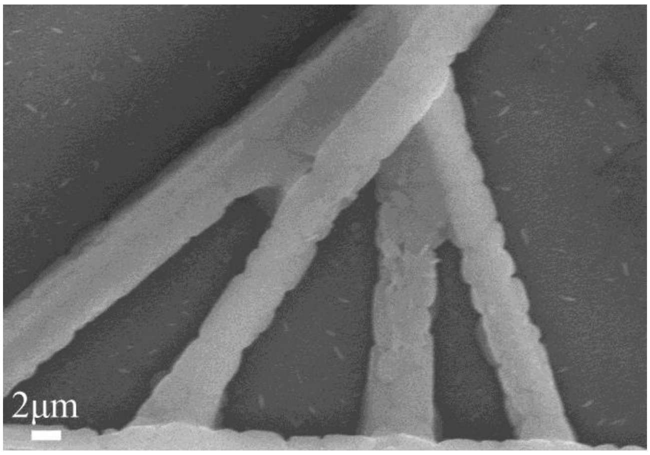

Figure S1. SEM images of perovskite $MAPbI_3$ nanowires formed by self-assemblyparticles.

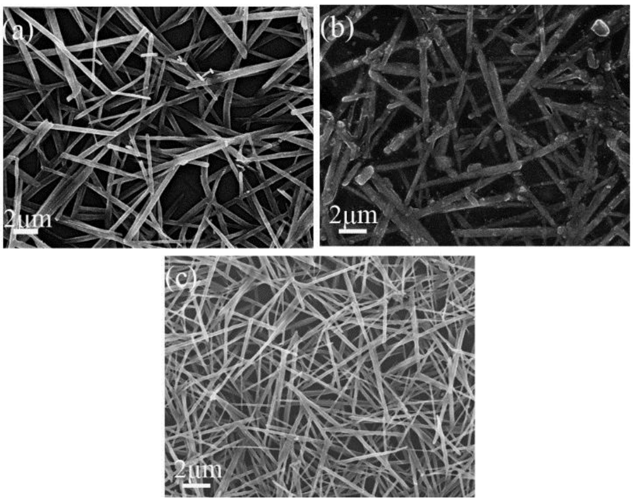

Figure S2. SEM images of perovskite $MAPbI_3$ nanowires with DMF volume of (a) 15 µL, (b) 10 µL, (c) 5 µL.

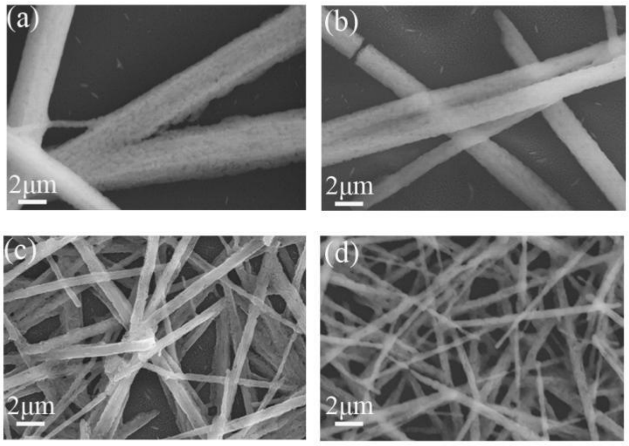

Figure S3. SEM images of perovskite $MAPbI_3$ nanowires with MAI concentration in IPA (a) 12.5 mg/5 mL, (b) 22.5 mg/5 mL, (c) 27.5 mg/5 mL, (d) 37.5 mg/5 mg.

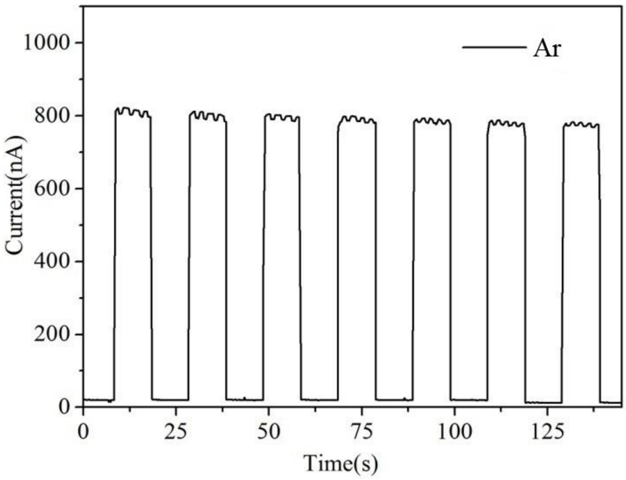

Figure S4. The I-t curves of devices fabricated under Ar and tested in inner atmosphere.

© 2018 by the authors. Licensee MDPI, Basel, Switzerland. This article is an open access article distributed under the terms and conditions of the Creative Commons Attribution (CC BY) license (http://creativecommons.org/licenses/by/4.0/).
